# Editorial: Cellular senescence in aging-related diseases: mechanisms and therapeutic targets

**DOI:** 10.3389/fcell.2026.1835072

**Published:** 2026-04-01

**Authors:** Jianjun Zhou, Honghe Wang, Fangfang Zhu

**Affiliations:** 1 Research Center for Translational Medicine, Cancer Stem Cell Institute, Shanghai East Hospital, School of Medicine, Tongji University, Shanghai, China; 2 Department of Biology and Center for Cancer Research, Tuskegee University, Tuskegee, AL, United States; 3 School of Biomedical Engineering, Shanghai Jiao Tong University, Shanghai, China; 4 HemaCell Biotechnology Inc., Suzhou, China

**Keywords:** aging-related diseases, cellular senescence, SASP, senescence-associated secretory phenotype, senolytics, therapeutic targets

Cellular senescence is a fundamental biological process characterized by the irreversible arrest of cell division, typically triggered by various stressors including telomere attrition, DNA damage, and oxidative stress. While this mechanism initially evolved as a potent tumor-suppressive barrier, the progressive accumulation of senescent cells during aging creates a significant pathological burden. These metabolically active cells secrete a complex cocktail of pro-inflammatory factors collectively known as the senescence-associated secretory phenotype (SASP), which drives chronic inflammation, disrupts tissue architecture, and promotes the development and progression of numerous age-related diseases. This Research Topic was conceived to delve into the intricate processes underlying cellular senescence, its multifaceted role in age-related pathology, and the development of novel therapeutic strategies to mitigate its deleterious effects. The articles collected here—comprising original research papers, comprehensive reviews, and clinical study protocol—span the spectrum from fundamental molecular mechanisms to translational applications, collectively advancing our understanding of senescence as both a driver of disease and a target for intervention.

The intricate interplay between cellular senescence and other fundamental biological processes is elegantly explored in several contributions. Ding et al. present a comprehensive review examining the paradoxical relationship between senescence and cellular reprogramming. They demonstrate that senescent cells, through their SASP, can paradoxically enhance the reprogramming efficiency of neighboring cells via paracrine factors such as IL-6, while unsuccessful reprogramming attempts can trigger senescence as a failsafe mechanism. This bidirectional crosstalk has profound implications for both regenerative medicine and cancer therapy. The cellular mechanisms of senescent cell clearance are addressed in the original research by Funk et al., who demonstrate that NK cells efficiently recognize and eliminate senescent renal tubular epithelial cells through NKG2D receptor engagement with ligands H60b and Mult-1. This clearance is perforin-dependent and significantly impaired by the immunosuppressive drug cyclosporine A, providing mechanistic insights into why senescent cells accumulate under immunosuppressive conditions. Liu et al. introduce an environmental dimension to senescence research, demonstrating how microplastics accelerate cellular aging through mitochondrial dysfunction, oxidative stress, and chronic inflammation, ultimately activating the cGAS-STING pathway and driving cellular senescence.

The organ-specific consequences of cellular senescence are explored across multiple disease contexts. Wu et al. provide a comprehensive review of how intrauterine growth restriction programs long-term cardiovascular vulnerability through mechanisms intimately linked to cellular senescence, including persistent alterations in endothelial cells, smooth muscle cells, and cardiomyocytes via oxidative stress, mitochondrial damage, and epigenetic modifications. In the metabolic domain, Kasperova et al. establish cellular senescence as both a cause and consequence of metabolic dysfunction, with visceral adipose tissue serving as a major source of SASP factors that propagate systemic inflammation and impair insulin signaling. They systematically review senescence accumulation in pancreatic β-cells, diabetic kidney disease, and cardiovascular complications, while critically evaluating the therapeutic potential of senolytics in metabolic disease. Musculoskeletal aging is addressed by Tian et al. through a systematic review demonstrating that whole-body vibration training significantly improves muscle strength in older adults, with effects mediated through enhanced neuromuscular junction transmission, improved muscle fiber capillarization, and modulation of protein synthesis/degradation balance. The complex relationship between senescence and cancer is explored by Zhang et al., who constructed a prognostic model based on necroptosis-related long non-coding RNAs that effectively predicts survival and stratifies prostate cancer patients into distinct immune phenotypes, with NR2F1-AS1 experimentally validated as an oncogenic driver.

The translational potential of senescence-targeting strategies is exemplified by Mu et al., who systematically optimized prime editing technology to achieve up to 80% editing efficiency across multiple cell lines, providing powerful tools for genetic interventions in age-related diseases. The clinical translation of senescence research is represented by Lu et al.'s study protocol investigating the comorbidity of postmenopausal osteoporosis and knee osteoarthritis, combining bone mineral density assessment, clinical evaluation, Traditional Chinese Medicine syndrome differentiation, and transcriptomic analysis to identify clinically relevant subtypes and inform personalized management strategies.

Collectively, the articles in this Research Topic advance our understanding of cellular senescence as a process deeply interconnected with reprogramming, immunity, metabolism, and regulated cell death. The context-dependence of senescence effects is paramount—the same SASP factors that promote tissue repair in acute settings drive pathology when chronically present. Methodological innovations, including single-cell transcriptomics and advanced *in vivo* models, are essential for translating mechanistic insights into clinical applications. As we look to the future, several research priorities emerge: (1) identifying cell-type-specific senescence programs and developing targeted elimination strategies; (2) understanding the interplay between senescence and other hallmarks of aging (for examples, mitochondrial dysfunction, epigenetic alterations, stem cell exhaustion); (3) developing biomarkers that reliably quantify senescent cell burden and predict therapeutic response; (4) conducting well-designed clinical trials to evaluate senotherapeutics in age-related diseases; and (5) exploring combination strategies that simultaneously target multiple aging pathways.

We are grateful to all authors for their valuable contributions and hope this Research Topic inspires further research into the mechanisms of cellular senescence and accelerates the development of interventions targeting this fundamental aging process, ultimately improving healthspan worldwide.

